# *MARCO* variants are associated with phagocytosis, pulmonary tuberculosis susceptibility and Beijing lineage

**DOI:** 10.1038/gene.2016.43

**Published:** 2016-11-17

**Authors:** N T T Thuong, T T B Tram, T D Dinh, P V K Thai, D Heemskerk, N D Bang, T T H Chau, D G Russell, G E Thwaites, T R Hawn, M Caws, S J Dunstan

**Affiliations:** 1 Oxford University Clinical Research Unit, Ho Chi Minh City, Vietnam; 2 Centre for Tropical Medicine and Global Health, Nuffield Department of Medicine, University of Oxford, Oxford, UK; 3 Pham Ngoc Thach Hospital for Tuberculosis and Lung Disease, Ho Chi Minh City, Vietnam; 4 Hospital for Tropical Diseases, Ho Chi Minh City, Vietnam; 5 Department of Microbiology and Immunology, College of Veterinary Medicine, Cornell University, Ithaca, NY, USA; 6 Department of Medicine, University of Washington School of Medicine, Seattle, WA, USA; 7 Liverpool School of Tropical Medicine, Pembroke Place, Liverpool, UK; 8 Peter Doherty Institute for Infection and Immunity, The University of Melbourne, Melbourne, Australia

## Abstract

Macrophage receptor with collagenous structure (MARCO) has an important role in the phagocytosis of *Mycobacterium tuberculosis* (*M. tuberculosis*). We hypothesized that *MARCO* polymorphisms are associated with phagocytosis, tuberculosis (TB) disease susceptibility and presentation, and infecting lineage. We used a human cellular model to examine how *MARCO* genotype mediates the immune response; a case–control study to investigate tuberculosis host genetic susceptibility; and a host–pathogen genetic analysis to study host–pathogen interactions. Two *MARCO* heterozygous (AG) genotypes (single-nucleotide polymorphisms rs2278589 and rs6751745) were associated with impaired phagocytosis of *M. tuberculosis* trehalose 6,6'-dimycolate-cord factor and β-glucan-coated beads in macrophages. The heterozygous genotypes of rs2278589 and rs6751745 were also associated with increased risk of pulmonary TB (PTB; rs2278589, *P*=0.001, odds ratio (OR)=1.6; rs6751745, *P*=0.009, OR=1.4), and with severe chest X-ray abnormalities (*P*=0.007, OR=1.6). These two genotypes were also associated with the Beijing lineage (rs2278589, *P*=0.001, OR=1.7; rs6751745, *P*=0.01, OR=1.5). Together, these results suggest that *MARCO* polymorphisms may regulate phagocytosis of *M. tuberculosis* and susceptibility and severity of PTB. They also suggest *MARCO* genotype and Beijing strains may interact to increase the risk of PTB.

## Introduction

Although tuberculosis (TB) can be cured, it is still one of the most devastating diseases, and globally causes active TB in 9.6 million and kills 1.5 million people annually.^1^ Variation in the host and pathogen are involved in disease susceptibility and determine disease development and outcome. Studies suggest that polymorphisms in host immunity genes influence susceptibility to TB,^2, 3^ especially in genes encoding Toll-like receptors, C-type lectin and scavenger receptors, which are involved in recognizing, binding and phagocytosing *Mycobacterium tuberculosis* (*M. tuberculosis*).

Scavenger receptors are cell surface receptors, which bind a variety of ligands, and have an important function in clearance of many foreign microorganisms. Class A and class B scavenger receptors are involved in the cytokine response to mycobacterial lipoarabinomannans^4^ and lipopeptides.^5^ Macrophage receptor with collagenous structure (MARCO) is a member of the class A scavenger receptor family. MARCO, on the cell surface of macrophages, binds bacteria to facilitate phagocytosis and activates immune responses.^6, 7, 8^ As such, MARCO-deficient mice have a reduced ability to clear bacteria in pneumonia.^7, 9^ Class A scavenger receptors and MARCO participate in phagocytosis of mycobacterial species, including *Mycobacterium leprae*,^10^ *Mycobacterium* *bovis* Bacille Calmette–Guérin,^11^ *Mycobacterium* *marinum*^6^ and *M. tuberculosis*.^12, 13^ More specifically, it has been demonstrated that *M. tuberculosis* is captured by MARCO *in vivo* via its cell wall cord factor (trehalose 6,6'-dimycolate or TDM), which increases pro-inflammatory cytokine response through the interaction with Toll-like receptors and CD14.^14^

The genetic diversity of *M. tuberculosis* is another factor that contributes to the clinical consequences of TB.^15, 16, 17^ The emergence of Beijing strains, which account for ~50% of strains in East Asia and 13% of strains worldwide,^18^ may contribute to disease susceptibility, drug resistance and treatment outcome. There is a possibility of human-mycobacterial co-evolution based on the genetic interactions of genes in the host and pathogen.^18, 19^ This would help to explain the interactions between host and pathogen factors in the development of TB.

Altogether, due to the role of phagocytosis and the potential function of MARCO in the immune response against *M. tuberculosis*, we hypothesized that (i) phagocytic activity is associated with developing different clinical phenotypes of TB, such as latent, pulmonary or extra-pulmonary TB; (ii) polymorphisms in *MARCO* regulate macrophage phagocytic activity; (iii) polymorphisms in *MARCO*, which contribute to the impairment of macrophage phagocytic activity, are associated with susceptibility to tuberculosis and influence clinical presentations and treatment failure; (iv) host and pathogen genotypes combined influence tuberculosis susceptibility.

## Results

### Phagocytosis and TB clinical phenotypes

**Figure 1:**
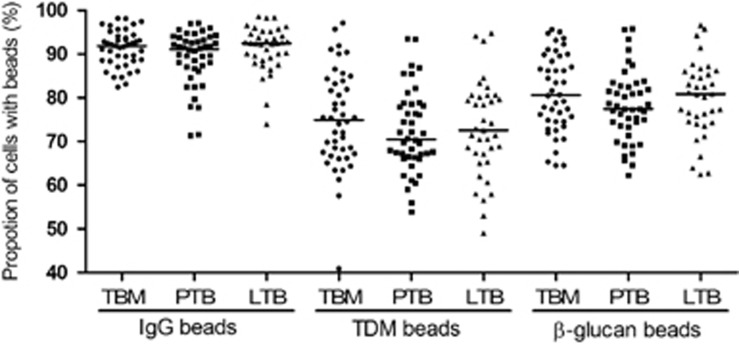
Phagocytic ability of macrophages from individuals with different TB phenotypes. Monocyte-derived macrophages from patients at day 7 were treated with Alexa 547-beads coated with either immunoglobulin-G (IgG), trehalose 6,6'-dimycolate (TDM) or β-glucan. Phagocytic ability was determined by the percentage of macrophages with beads in three TB phenotypes (55 TB meningitis, 52 pulmonary TB and 56 latent TB). Bars in plots represent median values. Comparisons across three groups of TB forms or genotypes were performed by using one-way analysis of variance. On these comparisons, *P*-values >0.05.

We examined phagocytosis in human monocyte-derived macrophages (MDMs) by bead-based internalization assays. Alexa 594-beads coated with immunoglobulin-G (IgG), TDM or β-glucan were added to MDMs and the percentage of macrophages with or without beads was measured using flow cytometry to assess phagocytic ability. Phagocytosis was assessed in macrophages isolated from patients with latent (*N*=56), pulmonary (*N*=52) or meningeal TB (*N*=55). No association was observed between phagocytic activity and different clinical forms of TB (Figure 1).

There was a wide range of phagocytic activities, with up to 50% of beads coated with *M. tuberculosis* TDM in macrophages from latent, pulmonary and meningeal TB (Figure 1). To investigate how MARCO influences the heterogeneity of phagocytic activity, we next examined the association of *MARCO* variants and phagocytosis.

### Association of MARCO SNPs with macrophage phagocytosis, mRNA expression and cytokines in response to *M. tuberculosis*

MARCO is a phagocytic receptor on macrophages which binds bacteria and facilitates phagocytosis to control and clear pathogens.^6, 8^ TDM from *M. tuberculosis* is a ligand of MARCO, whereas β-glucan is not known to be a MARCO ligand. Scavenger receptors on human monocytes have been found to bind to β-glucan,^20^ and MARCO (on CpG-ODN-pretreated macrophages) has been found to participate in the uptake of zymosan (which is derived from β-glucan);^21^ therefore β-glucan was used in this study to address the question of whether it might be a ligand for MARCO and to explore possible interaction between MARCO, tuberculosis and β-glucan.

**Figure 2:**
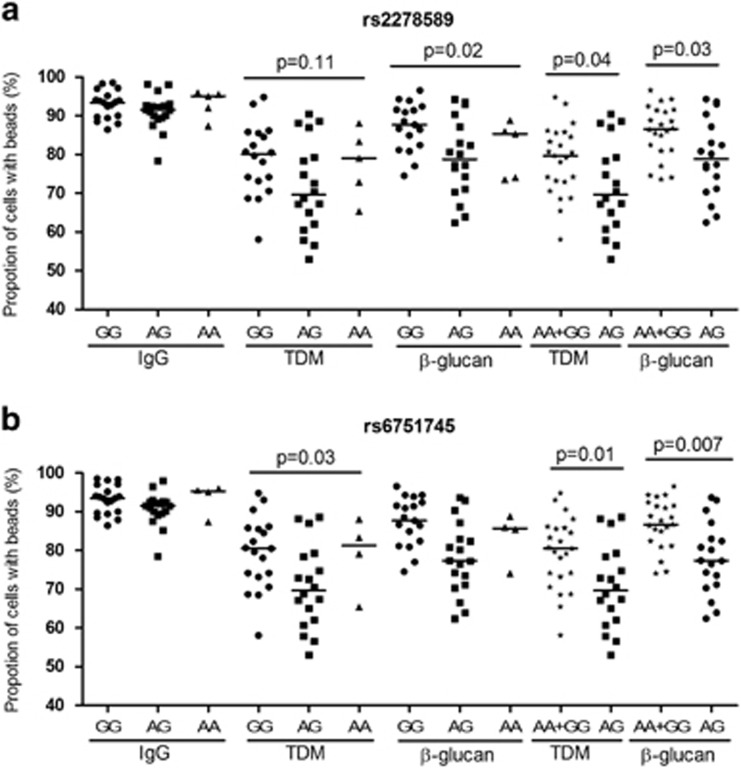
Phagocytic ability of macrophages from healthy subjects. Macrophage phagocytosis of beads was assessed according to *MARCO* SNP genotypes in healthy subjects; (**a**) rs2278589 (18 GG, 18 AG, 5 AA) and (**b**) rs6751745 (19 GG, 18 AG, 4 AA). Bars in plots represent median values. Comparisons across three groups of TB forms or genotypes were performed by using one-way analysis of variance, or two groups by using Mann–Whitney *U-*test.

We genotyped 12 *MARCO* haplotype-tagging single-nucleotide polymorphisms (SNPs) from 41 healthy subjects and performed phagocytosis assays. The genotypes of two SNPs, rs2278589 and rs6751745, were associated with phagocytosis of either TDM or β-glucan beads, but were not associated with phagocytosis of IgG beads (Figures 2a and b). The remaining 10 SNPs in *MARCO* were not associated with phagocytosis of any beads (Supplementary Figure S1). Furthermore, the results show the heterozygous genotypes of both SNPs were associated with reduced phagocytosis of TDM and β-glucan beads (rs2278589, *P*=0.04 and 0.03; rs6751745, *P*=0.01 and 0.007; Figures 2a and b).

**Figure 3:**
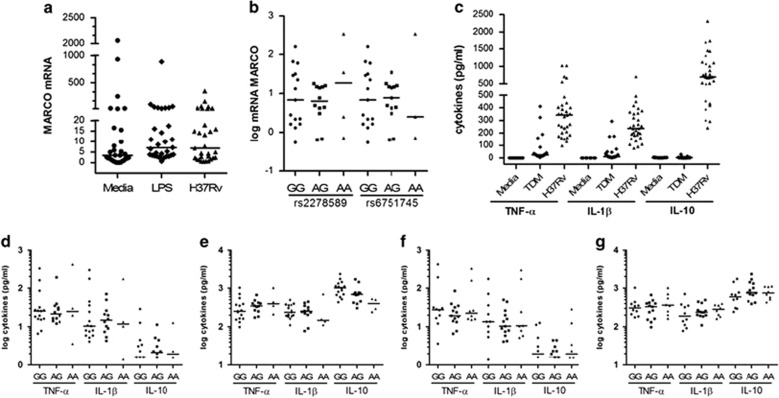
MARCO polymorphisms and variation in mRNA expression or cytokine production from healthy subjects. (**a**) mRNA was isolated from monocytes stimulated with Media, LPS at 100 ng ml^−1^ or *M. tuberculosis* whole-cell lysate at 5 μg ml^−1^. MARCO mRNA expression was measured and normalizes to glyceraldehyde-3-phosphate dehydrogenase. (**b**) Association of MARCO mRNA expression from cells stimulated with *M. tuberculosis* whole-cell lysate was analyzed with SNPs in *MARCO*: rs2278589 (4 AA, 12 AG, 15 GG), *P*=0.068 and rs6751745 (3 AA, 13 AG, 15 GG), *P*=0.039. (**c**) Cytokines were measured from monocytes stimulated with media, TDM at 100 μg ml^−1^ or *M. tuberculosis* whole-cell lysate at 25 μg ml^−1^. Cytokines from cells stimulated with TDM (**d**) or *M. tuberculosis* whole-cell lysate (**e**) were analyzed with SNP rs2278589 (4 AA, 12 AG, 15 GG). Cytokines from cells stimulated with TDM (**f**) or *M. tuberculosis* whole-cell lysate (**g**) were analyzed with SNP rs6751745 (3 AA, 13 AG, 15 GG). Data were collected from duplicate samples. Bars in plots represent median values. Comparisons across three genotypes were performed by using one-way analysis of variance.

We also examined the association between *MARCO* SNPs rs2278589 and rs6751745 and messenger RNA (mRNA) expression or cytokines in Peripheral blood mononuclear cells (PBMCs) from 31 healthy subjects. *MARCO* mRNA levels were up-regulated approximately twofold in PBMCs stimulated with lipopolysaccharide (LPS) or *M. tuberculosis* whole-cell lysate compared with unstimulated cells (Figure 3a). The genotypes of rs2278589 and rs6751745 were marginally associated with MARCO mRNA expression in cells stimulated with *M. tuberculosis* (Figure 3b; analysis of variance, *P*=0.068 and 0.039, respectively). For the heterozygous model, the AG genotype of these two SNPs was not significantly associated with reduced levels of MARCO mRNA in PBMCs stimulated with *M. tuberculosis*. For cytokine production, PBMCs were activated and produced pro-inflammatory cytokines TNF-α and IL-1β in response to both TDM and *M. tuberculosis* lysate. The anti-inflammatory cytokine IL-10 was induced by *M. tuberculosis* lysate stimulation, but not TDM (Figure 3c). In TDM or *M. tuberculosis* lysate stimulated cells, there was no association between the two SNP genotypes and TNF-α, IL-1β and IL-10 levels (Figures 3d and e for rs2278589, and Figures 3f and g for rs6751745).

Collectively, these data showed that the AG genotype of rs2278589 and rs6751745 in *MARCO* was not associated with *MARCO* mRNA expression or cytokine concentrations in PBMCs, but it was associated with reduced phagocytosis activated via TDM and β-glucan in macrophages.

### MARCO polymorphisms are associated with susceptibility to pulmonary TB, but not with TB meningitis

We used a case–control study to determine whether *MARCO* polymorphisms SNPs rs2278589 and rs6751745 are associated with susceptibility to TB, as macrophages with the heterozygote genotypes of these SNPs displayed reduced phagocytosis of *M. tuberculosis* ligands (Figure 2). Therefore we applied the heterozygote advantage model to analyze the relationship between *MARCO* SNPs and clinical TB, both pulmonary and meningeal. The heterozygote genotypes of rs2278589 and rs6751745 are associated with susceptibility to pulmonary tuberculosis (PTB; rs2278589; *P*=0.001, odds ratio (OR)=1.6 and rs6751745; *P*=0.009, OR=1.4; Table 1) and Supplementary Figure S2 shows that the two SNPs are in high linkage disequilibrium (LD) (*D*'=1, *r*^2^=0.88) in our Vietnamese Kinh control population. Associations between these two SNPs and PTB remained significant after Bonferroni correction (*P-*values × 2; Table 1).

Interestingly, the genotype frequencies of these two SNPs were different between PTB and tuberculous meningitis (TBM) under the heterozygote advantage model ((rs2278589; PTB 0.55, TBM 0.46; *P*=0.005, OR=1.4) (rs6751745; PTB 0.50, TBM 0.40; *P*=0.003, OR=1.5)). However the genotype frequencies of rs2278589 and rs6751745 in TBM patients were not different compared to the control groups using the genotypic model (*P*>0.05).

To thoroughly examine the association between *MARCO* SNPs and TB a further 10 SNPs, within and upstream of the *MARCO* gene, were analyzed. Apart from the two associated SNPs described above, rs6748401 (1.5 kb upstream) was associated with PTB in a genotypic comparison (*P*=0.039; Table 2), and none of others were associated with susceptibility to TB.

Collectively, two SNPs in the *MARCO* gene were associated with PTB, but not with TBM. The heterozygote genotypes of rs2278589 and rs6751745, which were associated with reduced phagocytic activity, were also associated with susceptibility to PTB.

### MARCO polymorphisms are associated with CXR presentation

To investigate whether *MARCO* polymorphisms influence clinical presentation or disease outcome, we examined the relationship between the two associated SNPs (rs2278589 and rs6751745), pre-treatment chest X-ray (CXR) abnormalities and 8-month treatment outcomes. Patients enrolled in this study were sputum smear-positive for PTB before treatment. Pre-treatment CXR showed 427/429 (99.5%) were abnormal with evidence of nodules (139, 32.4%), infiltrates (407, 94.9%), consolidation (40, 9.3%), cavities (139, 32.4%) and miliary disease (0, 0%).

SNPs rs2278589 and rs6751745 were associated with severity of CXR abnormality. SNP rs2278589 was associated with intermediate and severe CXR abnormality in the heterozygote model (*P*=0.008 intermediate; *P*=0.007 severe, OR=1.6; Table 3). SNP rs6751745 was associated with severe CXR abnormality in the heterozygote model (*P*=0.007, OR=1.6; Table 3).

There was no association between rs2278589 and rs6751745 genotype and poor treatment outcome (29/429, 6%), which was defined by death, or failure to convert to sputum smear negativity, however this may be due to the lack of events in this data set and consequent lack of power.

### MARCO polymorphisms are associated with Beijing lineage

Our previous studies reported associations between lineages of *M. tuberculosis*, particularly the modern Beijing lineage, and TB clinical phenotypes.^15, 17^ Given a worldwide emergence of the *M. tuberculosis* Beijing strains,^18^ we hypothesized that variation in the scavenger receptor MARCO, which binds to *Mycobacterium* and enables phagocytosis by macrophages, might be preferentially associated with a specific lineage. Therefore, we next examined whether the rs2278589 and rs6751745 genotypes are associated with infection caused by a particular bacterial lineage and whether this relationship influences disease phenotype.

The genotypic frequencies of rs2278589 and rs6751745 in all PTB patients (*N*=445) and in those patients where the lineage of the infecting *M. tuberculosis* isolate was determined (*N*=370), were compared with controls (Table 4). There was no significant association between the two SNPs and infection with either Indo-Oceanic or Euro-American lineage, or when combined as non-Beijing lineages. However, we found a significant association between these SNPs and infection with Beijing isolates in a genotypic comparison (rs2278589, *P*=0.005; rs6751745, *P*=0.033; Table 4), and in a heterozygous model (rs2278589, *P*=0.001, OR=1.7; rs6751745, *P*=0.012, OR=1.5; Table 4).

## Discussion

The primary finding of our study was that *MARCO* genotypes were associated with a reduction of phagocytosis of beads coated with pathogen-derived ligands, TDM from *M. tuberculosis*. These genotypes were also associated with increased susceptibility to PTB and severe chest radiography abnormality. Our results suggest that these polymorphisms may regulate phagocytosis of *M. tuberculosis*, and impairment of phagocytic ability could increase susceptibility to, and severity of, PTB. The *MARCO* genotypes were preferentially associated with Beijing rather than Indo-Oceanic or Euro-American lineages, which implies *MARCO* genotype may increase susceptibility to tuberculosis particularly of the Beijing lineage.

MARCO has a key role in bacterial phagocytosis and clearance.^6, 7, 9^ Recognition of TDM by MARCO, in conjunction with TLR2/CD4, activates transcriptional expression of immunity genes^22^ and cytokine production.^14^ However, no studies have yet shown the influence of *MARCO* genetic variation on the antimicrobial activity of macrophages such as phagocytosis or immune response. In this study, we found that *MARCO* SNPs rs2278589 and rs6751745 were not associated with gene expression and cytokine production in PBMCs but were associated with reduced phagocytosis of beads coated with pathogen-derived ligands, TDM or β-glucan in macrophages. We found that *MARCO* polymorphisms were not associated with cytokine production. In murine studies, MARCO-deficient macrophages were associated with a reduction of TNFα, IL-6 and IL-1β cytokine production in macrophages from MARCO knockout mice. The difference in study design could account for the differences seen in cytokine production between these two studies. We used human PBMCs, whereas in Bowdish *et al.*^14^ murine macrophages were used. In the macrophages from knockout mice, MARCO was absent, potentially having a major impact. In our study, MARCO was still produced, albeit a variant of MARCO with an unknown and potentially smaller impact. Another reason for the difference may be the limited numbers of samples in our study once stratified by genotype.

MARCO is involved in phagocytosis of bacteria, a step in pathogenesis that may be important in the development of PTB in the early phase of infection. The heterozygous genotypes of two *MARCO* SNPs were associated with reduced macrophage phagocytic function. The impairment of phagocytosis at the beginning of infection reduces the number of macrophages infected with *M. tuberculosis*, which then limits microbial killing and antigen presentation to lymphocytes.^23, 24^ The consequence of this could be the inadequate induction of innate and adaptive immune responses against *M. tuberculosis*, potentially increasing susceptibility to active disease. Deficient responses could also lead to increased microbial replication, which could manifest as severe abnormalities on CXR, such as was observed in TB patients carrying the heterozygous genotypes. Together, our data suggest that TB susceptibility and disease severity in patients with the *MARCO* AG genotype may be due to impairment of *M. tuberculosis* phagocytosis.

Our results show that variation in human *MARCO* is associated with susceptibility to PTB in the Vietnamese Kinh population. The associated intronic SNPs rs6748401 and rs2278589 are part of a wide haplotype block, suggesting they are markers in high LD with the unknown causative SNP(s). Two *MARCO* SNPs (rs17009726 or rs4491733) were previously associated with TB susceptibility in the Han Chinese Beijing and Gambian populations;^25, 26^ however, we did not observe any association with these SNPs in our TB population. Conversely, the associated SNPs (rs6748401 and rs2278589) described in this study were not associated in the Gambian population and were not genotyped in the Chinese population. The discrepancy in our results may be due to different population LD structure. The frequencies of the associated SNPs found in the three studies were very different based on the 1000 Genomes Project (http://www.ncbi.nlm.nih.gov/projects/SNP; rs17009726 minor allele frequency in African 0.0008, Ad Mixed American 0.0014, European 0.0060, East Asian 0.1210 and South Asian 0.1483 super populations) and overall linkage (*D*' plots) across the *MARCO* SNPs in three populations are visually different (Supplementary Figure S2; Bowdish *et al.*^25^and Ma *et al.*^26^). LD in the Vietnamese Kinh population across this gene region contains larger haplotype blocks with more SNPs compared with both the Han Chinese (HCB) and Gambian populations. The differing population structures in this gene region may account for the inability to replicate individual SNP associations, however the accumulated evidence across these populations suggests that *MARCO* variation contributes to PTB susceptibility.

The remarkable emergence of Beijing lineage worldwide, including Vietnam, supports the hypothesis that the variation in the scavenger receptor *MARCO*, which binds *Mycobacterium* and promotes macrophage internalization, might support the emergence of the Beijing lineages. Our data show associations of both *MARCO* variants and *M. tuberculosis* lineage with TB susceptibility suggesting potential for host–pathogen co-evolution, as reported previously with *TLR2*, *NRAMP1* and *EREG*.^15, 27, 28^ Our associated SNPs may be markers of non-synonymous structural variants of MARCO that effects ability to bind ligands from Beijing lineage strains, reducing phagocytosis and increasing susceptibility to TB. Host–pathogen co-evolution in tuberculosis needs to be studied on a larger scale with respect to patients and genes, coupled with functional studies to determine the underlying mechanisms.

The role of MARCO in macrophage phagocytosis is important in clearance of pathogens. Our results suggest that *MARCO* polymorphisms may regulate phagocytosis of *M. tuberculosis* and thus influence susceptibility to and severity of pulmonary tuberculosis. The results also suggest that *MARCO* genotype and Beijing strains may interact to increase the risk of pulmonary tuberculosis.

## Materials and methods

### Cellular studies

#### *Ex vivo* isolation of PBMCs and MDMs

PBMCs were separated from heparinized whole blood by Lymphoprep (Axis-Shield, Oslo, Norway) gradient centrifugation according to the manufacturer's protocol. From 20 ml of blood, we obtained ~1–1.5 × 10^7^ PBMCs. To isolate monocytes by adherence, PBMCs were plated in cell-culture-treated 48-well plates (Nunc, Roskilde, Denmark) with 9x10^5^ cells per well in media without serum, containing RPMI-1640 (Sigma, Munich, Germany), 2 mm l-glutamine and 100 units of penicillin. Cells were incubated at 37 °C, 5% CO_2_ for 2 h and the non-adhered cells were washed off gently two times by warm phosphate-buffered saline (PBS) with 3% fetal calf serum (Sigma). Cells were re-suspended in 0.4 ml complete media, containing RPMI-1640, 2 mM l-glutamine, 100 units of penicillin, 10% fetal calf serum and 10 ng ml^−1^ human mCSF (R&D Systems, Minneapolis, MN, USA), and incubated at 37 °C, 5% CO_2_. To derive monocytes, adhered cells were incubated for 5–7 days. Complete media was changed at day 4, and phagocytosis assay was performed at day 7.

#### PBMC stimulation for MARCO mRNA and cytokine measurement

For MARCO mRNA expression, PBMCs were plated in 24-well plates (10^6^ cells per well). Cells were subsequently stimulated with either whole-cell lysate of *M. tuberculosis* H37Rv at 5 μg ml^−1^ (Colorado State University, USA), LPS at 100 ng ml^−1^ (Sigma-Aldrich, St Louis, MO, USA) or media. For cytokine measurement, PBMCs were plated in 96-well plates (10^5^ cells per well) and then stimulated with either whole-cell lysate at 25 μg ml^−1^, TDM at 100 μg ml^−1^ (Enzo Life Sciences, Farmingdale, NY, USA), LPS 100 ng ml^−1^ or media. For stimulation, TDM was coated on the plate and air-dried in the cabinet the day before, while other ligands were added directly into media after cell plating.

For real-time quantitative PCR (RT-qPCR), after stimulation for 5 h, RNA was extracted by Trizol according to the manufacturer's protocol (Invitrogen, Carlsbad, CA, USA), dissolved in RNase-free water and stored at −70 °C until use. Taqman real-time PCR was performed to measure the expression level of *MARCO* gene using primers forward 5′-GGGCTCACCTGGTGGTTATC-3′ and reverse 5′-TCGACAACCTGGTCTGACAGT-3′ (Sigma-Aldrich Pte Ltd, Singapore), and probe 5′-Cyan500-CTCCGGGTCCTGGAGATGTATTTCCTCA-BHQ1-3′ (TIB MOLBIOL Syntheselabor GmbH, Berlin, Germany). Samples were normalized to *glyceraldehyde-3-phosphate dehydrogenase* and analyzed by using LightCycler 480 release 1.5.1.62 Relative Quantification software (Roche, Ho Chi Minh City, Vietnam).

For cytokine measurement, after stimulation for 24 h, supernatants were collected for cytokine measurement, including TNF-α, IL-1β and IL-10. Cytokine levels were determined with a sandwich ELISA technique by the Luminex multiplex bead array technology (Bio-Rad Laboratories, Inc., Hercules, CA, USA).

#### Preparation of beads for phagocytosis

The procedure of coating beads was adopted from Yates *et al.*^29^ Carboxylate-modified silica particles (25 mg or 500 μl of 3 μm; Kisker Biotech, Steinfurt, Germany) were washed three times in 1 ml of PBS by vortexing and centrifugation at 2000 *g* for 1 min. Beads were incubated at room temperature in 25 mg ml^−1^ cyanamide (Sigma-Aldrich), which works as a cross-linker, in PBS with agitation for 15 min. Beads were washed twice in 1 ml of coupling buffer (0.1 m borate buffer, pH 8.0) and then incubated in 0.5 ml coupling buffer with 1.0 mg defatted bovine serum albumin (Sigma-Aldrich) and 0.1 mg human IgG (Molecular Probes, Eugene, OR, USA) or 0.25 mg ligands (TDM (Enzo Life Sciences) or β-glucan/whole glucan particles (Invivogen, San Diego, CA, USA)) then dispersed for 12 h with agitation. The coated beads were washed three times in 1 ml of quench buffer (250 mM glycine, Sigma-Aldrich) to quench unreacted cyanamide. The beads were re-suspended in 1 ml coupling buffer with 10 μl of 5 mg ml^−1^ Alexa Fluor 594 succidinimyl ester (Molecular Probes) in dimethyl sulfoxide (DMSO, Sigma-Aldrich) and agitated for 1 h. The beads were washed three times in 1 ml quench buffer, re-suspended in 1 ml PBS with 0.02% sodium azide and stored at 4 °C.

#### Phagocytosis assays

At day 7, MDMs from healthy subjects or TB patients were checked by microscope to ensure a monolayer with 80–90% confluence had been achieved. After removing media, new media was added immediately with 200 μl per well in the 48-well plates. Stored beads were washed three times in PBS, then 10 μl of beads coated with Alexa Fluor 594 SE and IgG/ligand was added into each well with concentration to achieve an average of 1–2 beads internalized per macrophage. Binding and uptake of the beads was performed by incubating of macrophages with a suspension of the beads at 37 °C, 5% CO_2_ for 10 min. Cells were washed three times in PBS with 3% FBS to remove unbound beads, harvested by scraping in cold PBS with 1% para-formaldehyde and transferred into a tube for flow cytometry analysis.

Phagocytosis of macrophages was determined by the percentage of phagocytes that have internalized beads coated with Alexa Fluor 594 SE. Samples were run using BD FACSCanto II and FACSDiva acquisition software (Becton Dickinson, San Diego, CA, USA), and fluorescence intensity analyzed using FlowJo analysis software (BD Biosciences, San Jose, CA, USA). Bead ingestion was confirmed by the presence of bright green fluorescence in the proteolysis assay (Supplementary Figure S3). The green fluorescence indicated that almost all of the MDM-associated beads were internalized following 10 min incubation.

### Haplotype-tagging SNPs

We identified haplotype-tagging SNPs from the Han Chinese in Beijing (CHB) population from the International HapMap Project (http://www.hapmap.org) and obtained functional information of SNPs from UCSC Genome Bioinformatics (http://genome.ucsc.edu/). We searched a region on chromosome 2p14.2 encompassing *MARCO* and 10 kb upstream for tagged SNPs using an *r*^2^ cutoff of 0.8 for linkage disequilibrium and a minor allele frequency cut-off of 10%. Haploview 4.2 (Broad Institute of MIT and Harvard, USA) was used to calculate *r*^2^ and *D*' for linkage disequilibrium. Twelve haplotype-tagging SNPs including one SNP in the promoter region (3 bp upstream) and one missense SNP (in exon 10) were genotyped. SNPs in the *MARCO* gene were genotyped using the GoldenGate genotyping assay (Illumina, San Diego, CA, USA).

### Human subjects

For cellular studies, in addition to the TBM and PTB patients described below, latent TB (LTB) subjects (*N*=56) were recruited from healthy Vietnamese volunteers working at Oxford University Clinical Research Unit (OUCRU), Vietnam. They were diagnosed for LTB infection using T-SPOT.TB test (Oxford Immunotec, Abingdon, UK). Another group of healthy Vietnamese volunteers (*N*=31) from OUCRU was enrolled in cellular studies in which we genotyped SNPs on *MARCO* and examined macrophage phagocytosis, mRNA expression and immune response.

The case–control genetics association study cases comprised of 900 HIV negative adults with TB treated from 2008 to 2011 for PTB (*N*=450), and in several clinical trials and observational studies from 2001 to 2013 for TBM (*N*=450). PTB patients were recruited through the network of district TB control units, and TBM patients were recruited from either Pham Ngoc Thach Hospital for Tuberculosis and Lung Disease or the Hospital for Tropical Diseases, in Ho Chi Minh City, Vietnam. PTB patients had acid fast bacilli in sputum and TBM patients had clinical meningitis in addition to specific clinical requirements that have been previously described.^30^ Population controls were 450 newborn babies born at Hung Vuong Obstetric Hospital, Ho Chi Minh City. Peripheral blood samples from TB patients or umbilical cord blood from babies were collected. All samples came from unrelated individuals who were ethnic Vietnamese Kinh.

Written informed consent was obtained from each volunteer. Protocols were approved by human subjects review committees at the Hospital for Tropical Diseases and Pham Ngoc Thach Hospital for TB and Lung Disease, HCMC, Vietnam. Ethical approval was also granted by the Oxford Tropical Research Ethics Committee (UK).

### Case–control genetic association study and quality checking

Fourteen genotyped SNPs were tested for Hardy–Weinberg equilibrium (HWE) in control subjects using a *χ*^2^-test. SNPs were excluded if they had >5% missing genotype calls, a minor allele frequency of <10% or a HWE *P-*value of <0.05.

### Chest radiography

CXR were examined at the time of TB diagnosis, with reports provided by clinicians from district TB control units. Abnormal features on a chest radiograph were recorded comprising of nodules, infiltrates, consolidation, cavities and miliary TB. To grade chest radiograph severity, the abnormal features were assessed and classified as mild if abnormal features were present in one lobe, intermediate if abnormal features were present in one lung, and severe if abnormal features were present in both lungs.

### Statistical analysis

Host genetic analysis was performed using a Chi-squared test with two degrees of freedom in genotypic comparisons, or one in genotypic models (that is, dominant, recessive or heterozygous advantage model). A significance threshold of *P*<0.05 was used. For multiple SNP comparisons, Bonferroni correction was applied.

Comparisons across three clinical groups (PTB, TBM and controls) or genotypes were performed by using one-way analysis of variance or two groups by using Mann–Whitney *U-*test. Analyses were performed using SPSS version 14.0, USA. mRNA, cytokine and phagocytosis graphs were generated using GraphPad Prism version 6.04 for Windows (GraphPad Software, La Jolla, CA, USA, www.graphpad.com).

## Figures and Tables

**Table 1:** *MARCO* SNPs rs2278589 and rs6751745 are associated with pulmonary TB

*SNP*	*rs2278589*			*rs6751745*		
Control genotype (11, 12, 22) (*N*, %)	194 (0.45)	190 (0.44)	48 (0.11)	210 (0.48)	181 (0.42)	43 (0.10)
PTB genotype (11, 12, 22) (*N*, %)	165 (0.37)	245 (0.55)	35 (0.08)	194 (0.43)	225 (0.50)	27 (0.06)
Genotypic (*P*, P*)	**0.004**	**0.008**		**0.011**	**0.022**	
Dominant (*P*, P*, OR (95% CI))	0.101	0.202	1.5 (0.9-2.3)	**0.035**	0.070	1.7 (1.0–2.8)
Recessive (*P*, P*, OR (95% CI))	**0.018**	**0.036**	0.7 (0.5–0.9)	0.146	0.292	0.8 (0.6–1.1)
Heterozygous (*P*, P*, OR (95% CI))	**0.001**	**0.002**	1.6 (1.2–2.0)	**0.009**	**0.018**	1.4 (1.1–1.8)

Abbreviations: OR (95% CI), odds ratio (95% confidence interval); SNP, single-nucleotide polymorphism.

1: majority allele; 2: minority allele; Dominant is the comparison of 22 vs (11+12).

*P*=*P-*value.

P*=corrected *P*-value, Bonferroni correction by two SNPs (*P*-values × 2). Bold entries indicate *P*-values <0.05.

**Table 2:** Summary of genotyped SNPs in *MARCO*

*rs ID* *Location*	*Cases*		*Controls*		*Genotypic*
	*PTB/TBM*	*11/12/22*	*11/12/22*	*HWE-*P	P*-values*
rs7573346	PTB	131/233/84	122/213/105	0.524	0.175
4.9 Kb upstream	TBM	127/216/101			0.913
**rs6748401**	PTB	108/245/90	120/215/100	0.845	**0.039**
1.5 Kb upstream	TBM	115/223/109			0.788
rs1318645	PTB	109/246/92	119/215/100	0.879	0.057
3 bp upstream	TBM	115/223/109			0.815
rs4491733	PTB	104/240/102	114/228/93	0.290	0.593
intron 1	TBM	120/222/101			0.782
rs12998782	PTB	228/184/34	243/160/31	0.510	0.345
intron 1	TBM	239/168/33			0.883
rs17009726	PTB	331/110/8	340/94/6	0.863	0.456
intron 1	TBM	342/99/5			0.911
**rs2278589**	PTB	165/245/35	194/190/48	0.885	**0.004**
intron 3	TBM	194/203/47			0.871
rs1371562	PTB	289/141/15	284/138/15	0.724	0.998
intron 6	TBM	286/140/15			0.998
rs6761637	PTB	323/114/8	335/89/9	0.289	0.202
exon 10	TBM	333/101/6			0.519
**rs6751745**	PTB	194/225/27	210/181/43	0.663	**0.011**
intron 13	TBM	223/178/39			0.752
rs17796260	PTB	293/139/13	283/135/18	0.708	0.622
intron 13	TBM	292/136/14			0.739
rs3765035	PTB	145/251/49	152/220/60	0.164	0.210
intron 15	TBM	183/199/60			0.149

Abbreviations: HWE, Hardy–Weinberg equilibrium; *P*, *P*-value; PTB, pulmonary tuberculosis; TBM, tuberculous meningitis.

1: majority allele; 2: minority allele. Bold values indicate the SNPs that have genotypic *P*-values <0.05.

**Table 3:** *MARCO* SNPs rs2278589 and rs6751745 are associated with level of CXR abnormality in PTB patients

*Abnormality level*	*Genotype*			*Genotypic*	*Heterozygous*	
	*GG*	*AG*	*AA*	P*-values*	P*-values*	*OR (95% CI)*
*rs2278589*						
Controls	194 (0.45)	190 (0.44)	48 (0.11)			
Mild	26 (0.38)	39 (0.57)	4 (0.06)	0.112	0.052	1.7 (1.0–2.7)
Intermediate	67 (0.37)	101 (0.56)	13 (0.07)	**0.022**	**0.008**	1.6 (1.1–2.3)
Severe	60 (0.35)	96 (0.56)	15 (0.09)	**0.026**	**0.007**	1.6 (1.1–2.3)
						
*rs6751745*						
Controls	210 (0.48)	181 (0.42)	43 (0.10)			
Mild	31 (0.44)	35 (0.50)	4 (0.06)	0.314	0.193	1.4 (0.8–2.3)
Intermediate	82 (0.45)	90 (0.50)	9 (0.05)	0.055	0.068	1.4 (1.0–2.0)
Severe	68 (0.40)	92 (0.54)	11 (0.06)	**0.026**	**0.007**	1.6 (1.1–2.3)

Bold entries indicate *P*-values <0.05.

**Table 4:** *MARCO* SNPs rs2278589 and rs6751745 are associated with the Beijing strain

*Group*	*Genotype*			*Genotypic*	*Heterozygous*	
	*GG*	*AG*	*AA*	P*-values*	P*-values*	*OR (95%CI)*
*rs2278589*						
Controls	194 (0.45)	190 (0.44)	48 (0.11)			
PTB	165 (0.37)	245 (0.55)	35 (0.08)	**0.004**	**0.001**	1.6 (1.2–2.0)
All isolates	135 (0.36)	205 (0.55)	30 (0.08)	**0.005**	**0.001**	1.6 (1.2–2.1)
Non-Beijing	61 (0.42)	77 (0.53)	8 (0.05)	0.060	0.066	1.4 (1.0–2.1)
East Asian/Beijing	74 (0.33)	128 (0.57)	22 (0.10)	**0.005**	**0.001**	1.7 (1.2–2.3)
						
*rs6751745*						
Controls	210 (0.48)	181 (0.42)	43 (0.10)			
PTB	194 (0.43)	225 (0.50)	27 (0.06)	**0.011**	**0.009**	1.4 (1.1–1.9)
All isolates	161 (0.47)	187 (0.48)	23 (0.05)	**0.021**	**0.014**	1.4 (1.1–1.9)
Non-Beijing	68 (0.47)	70 (0.48)	8 (0.05)	0.174	0.188	1.3 (0.9–1.9)
East Asian/Beijing	93 (0.41)	117 (0.52)	15 (0.07)	**0.033**	**0.012**	1.5 (1.1–2.1)

Bold entries indicate *P*-values <0.05.

## References

[bib1] WHO Gobal Tuberculosis Report 2015 20th edn WHO: Geneva, Switzerland, 2015.

[bib2] Berrington WR, Hawn TR. *Mycobacterium tuberculosis*, macrophages, and the innate immune response: does common variation matter? Immunol Rev 2007; 219: 167–186. 17850489 10.1111/j.1600-065X.2007.00545.x PMC2859969

[bib3] Kleinnijenhuis J, Oosting M, Joosten LA, Netea MG, Van Crevel R. Innate immune recognition of *Mycobacterium tuberculosis*. Clin Dev Immunol 2011; 2011: 405310. 21603213 10.1155/2011/405310 PMC3095423

[bib4] Jozefowski S, Sobota A, Pawlowski A, Kwiatkowska K. Mycobacterium tuberculosis lipoarabinomannan enhances LPS-induced TNF-alpha production and inhibits NO secretion by engaging scavenger receptors. Microb Pathog 2011; 50: 350–359. 21419839 10.1016/j.micpath.2011.03.001

[bib5] Drage MG, Pecora ND, Hise AG, Febbraio M, Silverstein RL, Golenbock DT et al. TLR2 and its co-receptors determine responses of macrophages and dendritic cells to lipoproteins of *Mycobacterium tuberculosis*. Cell Immunol 2009; 258: 29–37. 19362712 10.1016/j.cellimm.2009.03.008 PMC2730726

[bib6] Benard EL, Roobol SJ, Spaink HP, Meijer AH. Phagocytosis of mycobacteria by zebrafish macrophages is dependent on the scavenger receptor Marco, a key control factor of pro-inflammatory signalling. Dev Comp Immunol 2014; 47: 223–233. 25086293 10.1016/j.dci.2014.07.022

[bib7] Dorrington MG, Roche AM, Chauvin SE, Tu Z, Mossman KL, Weiser JN et al. MARCO is required for TLR2- and Nod2-mediated responses to *Streptococcus pneumoniae* and clearance of pneumococcal colonization in the murine nasopharynx. J Immunol 2013; 190: 250–258. 23197261 10.4049/jimmunol.1202113 PMC3529821

[bib8] Thelen T, Hao Y, Medeiros AI, Curtis JL, Serezani CH, Kobzik L et al. The class A scavenger receptor, macrophage receptor with collagenous structure, is the major phagocytic receptor for *Clostridium sordellii* expressed by human decidual macrophages. J Immunol 2010; 185: 4328–4335. 20810988 10.4049/jimmunol.1000989 PMC7682803

[bib9] Arredouani M, Yang Z, Ning Y, Qin G, Soininen R, Tryggvason K et al. The scavenger receptor MARCO is required for lung defense against pneumococcal pneumonia and inhaled particles. J Exp Med 2004; 200: 267–272. 15263032 10.1084/jem.20040731 PMC2212010

[bib10] Montoya D, Cruz D, Teles RM, Lee DJ, Ochoa MT, Krutzik SR et al. Divergence of macrophage phagocytic and antimicrobial programs in leprosy. Cell Host Microbe 2009; 6: 343–353. 19837374 10.1016/j.chom.2009.09.002 PMC2764558

[bib11] Haworth R, Platt N, Keshav S, Hughes D, Darley E, Suzuki H et al. The macrophage scavenger receptor type A is expressed by activated macrophages and protects the host against lethal endotoxic shock. J Exp Med 1997; 186: 1431–1439. 9348300 10.1084/jem.186.9.1431 PMC2199123

[bib12] Pedroza-Gonzalez A, Garcia-Romo GS, Aguilar-Leon D, Calderon-Amador J, Hurtado-Ortiz R, Orozco-Estevez H et al. *In situ* analysis of lung antigen-presenting cells during murine pulmonary infection with virulent *Mycobacterium tuberculosis*. Int J Exp Pathol 2004; 85: 135–145. 15255967 10.1111/j.0959-9673.2004.00381.x PMC2517470

[bib13] Zimmerli S, Edwards S, Ernst JD. Selective receptor blockade during phagocytosis does not alter the survival and growth of *Mycobacterium tuberculosis* in human macrophages. Am J Respir Cell Mol Biol 1996; 15: 760–770. 8969271 10.1165/ajrcmb.15.6.8969271

[bib14] Bowdish DM, Sakamoto K, Kim MJ, Kroos M, Mukhopadhyay S, Leifer CA et al. MARCO, TLR2, and CD14 are required for macrophage cytokine responses to mycobacterial trehalose dimycolate and *Mycobacterium tuberculosis*. PLoS Pathog 2009; 5: e1000474. 19521507 10.1371/journal.ppat.1000474 PMC2688075

[bib15] Caws M, Thwaites G, Dunstan S, Hawn TR, Lan NT, Thuong NT et al. The influence of host and bacterial genotype on the development of disseminated disease with *Mycobacterium tuberculosis*. PLoS Pathog 2008; 4: e1000034. 18369480 10.1371/journal.ppat.1000034 PMC2268004

[bib16] Gagneux S, Small PM. Global phylogeography of *Mycobacterium tuberculosis* and implications for tuberculosis product development. Lancet Infect Dis 2007; 7: 328–337. 17448936 10.1016/S1473-3099(07)70108-1

[bib17] Thwaites G, Caws M, Chau TT, D'Sa A, Lan NT, Huyen MN et al. Relationship between *Mycobacterium tuberculosis* genotype and the clinical phenotype of pulmonary and meningeal tuberculosis. J Clin Microbiol 2008; 46: 1363–1368. 18287322 10.1128/JCM.02180-07 PMC2292951

[bib18] Parwati I, van Crevel R, van Soolingen D. Possible underlying mechanisms for successful emergence of the *Mycobacterium tuberculosis* Beijing genotype strains. Lancet Infect Dis 2010; 10: 103–111. 20113979 10.1016/S1473-3099(09)70330-5

[bib19] Gagneux S. Host-pathogen coevolution in human tuberculosis. Philos Trans R Soc Lond B Biol Sci 2012; 367: 850–859. 22312052 10.1098/rstb.2011.0316 PMC3267123

[bib20] Rice PJ, Kelley JL, Kogan G, Ensley HE, Kalbfleisch JH, Browder IW et al. Human monocyte scavenger receptors are pattern recognition receptors for (1—>3)-beta-D-glucans. J Leukoc Biol 2002; 72: 140–146. 12101273

[bib21] Jozefowski S, Yang Z, Marcinkiewicz J, Kobzik L. Scavenger receptors and beta-glucan receptors participate in the recognition of yeasts by murine macrophages. Inflamm Res 2012; 61: 113–126. 22116297 10.1007/s00011-011-0395-5 PMC3265724

[bib22] Sakamoto K, Kim MJ, Rhoades ER, Allavena RE, Ehrt S, Wainwright HC et al. Mycobacterial trehalose dimycolate reprograms macrophage global gene expression and activates matrix metalloproteinases. Infect Immun 2013; 81: 764–776. 23264051 10.1128/IAI.00906-12 PMC3584883

[bib23] Fujiwara N, Kobayashi K. Macrophages in inflammation. Curr Drug Targets Inflamm Allergy 2005; 4: 281–286. 16101534 10.2174/1568010054022024

[bib24] Russell DG, Vanderven BC, Glennie S, Mwandumba H, Heyderman RS. The macrophage marches on its phagosome: dynamic assays of phagosome function. Nat Rev Immunol 2009; 9: 594–600. 19590530 10.1038/nri2591 PMC2776640

[bib25] Bowdish DM, Sakamoto K, Lack NA, Hill PC, Sirugo G, Newport MJ et al. Genetic variants of MARCO are associated with susceptibility to pulmonary tuberculosis in a Gambian population. BMC Med Genet 2013; 14: 47. 23617307 10.1186/1471-2350-14-47 PMC3652798

[bib26] Ma MJ, Wang HB, Li H, Yang JH, Yan Y, Xie LP et al. Genetic variants in MARCO are associated with the susceptibility to pulmonary tuberculosis in Chinese Han population. PLoS One 2011; 6: e24069. 21886847 10.1371/journal.pone.0024069 PMC3160327

[bib27] van Crevel R, Parwati I, Sahiratmadja E, Marzuki S, Ottenhoff TH, Netea MG et al. Infection with *Mycobacterium tuberculosis* Beijing genotype strains is associated with polymorphisms in SLC11A1/NRAMP1 in Indonesian patients with tuberculosis. J Infect Dis 2009; 200: 1671–1674. 19863441 10.1086/648477

[bib28] Thuong NT, Hawn TR, Chau TT, Bang ND, Yen NT, Thwaites GE et al. Epiregulin (EREG) variation is associated with susceptibility to tuberculosis. Genes Immun 2012; 13: 275–281. 22170233 10.1038/gene.2011.83 PMC3684976

[bib29] Yates RM, Hermetter A, Russell DG. Recording phagosome maturation through the real-time, spectrofluorometric measurement of hydrolytic activities. Methods Mol Biol 2009; 531: 157–171. 19347317 10.1007/978-1-59745-396-7_11 PMC2756812

[bib30] Thwaites GE, Nguyen DB, Nguyen HD, Hoang TQ, Do TT, Nguyen TC et al. Dexamethasone for the treatment of tuberculous meningitis in adolescents and adults. N Engl J Med 2004; 351: 1741–1751. 15496623 10.1056/NEJMoa040573

