# Supplementary material for: MARCO variants are associated with phagocytosis, pulmonary tuberculosis susceptibility and Beijing lineage
Source: Genes Immun. 2016 Nov 17;17(7):419–25. doi: 10.1038/gene.2016.43 (PMC5133378; doi:10.1038/gene.2016.43)
Supplement: Supplementary Figures [file gene201643x1.docx]

**Supplementary Figures**

**
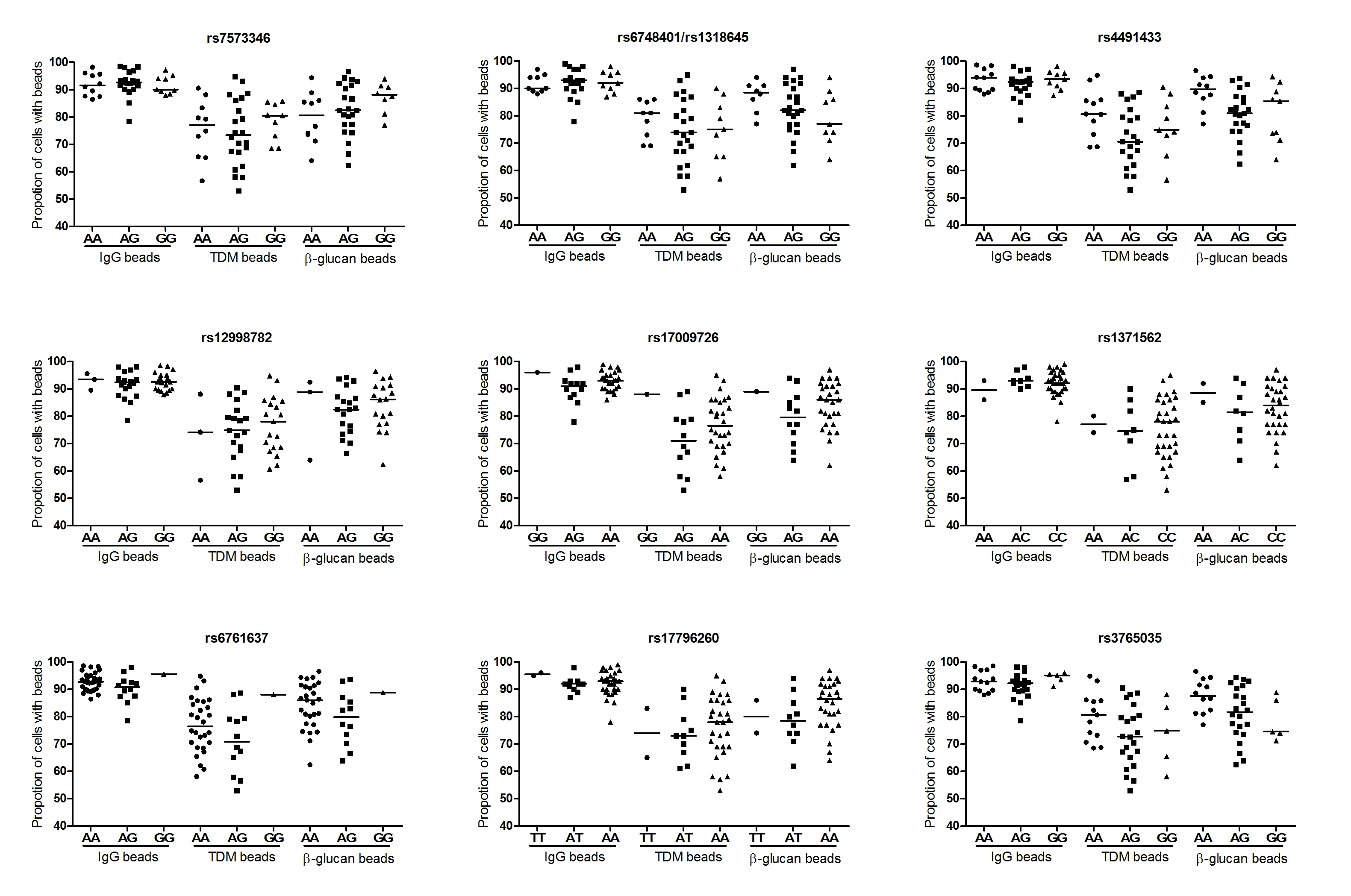
**

**Figure S1. Phagocytic ability of macrophages from healthy subjects**

Macrophage phagocytosis of IgG, TDM and β-glucan beads was plotted by *MARCO* SNP genotype. Data is shown for 10 tagging-SNPs in 41 healthy subjects. Bars in plots represent median values. Comparisons across three groups of TB forms or genotypes were performed by using one-way ANOVA. For all these comparisons the p value > 0.05.


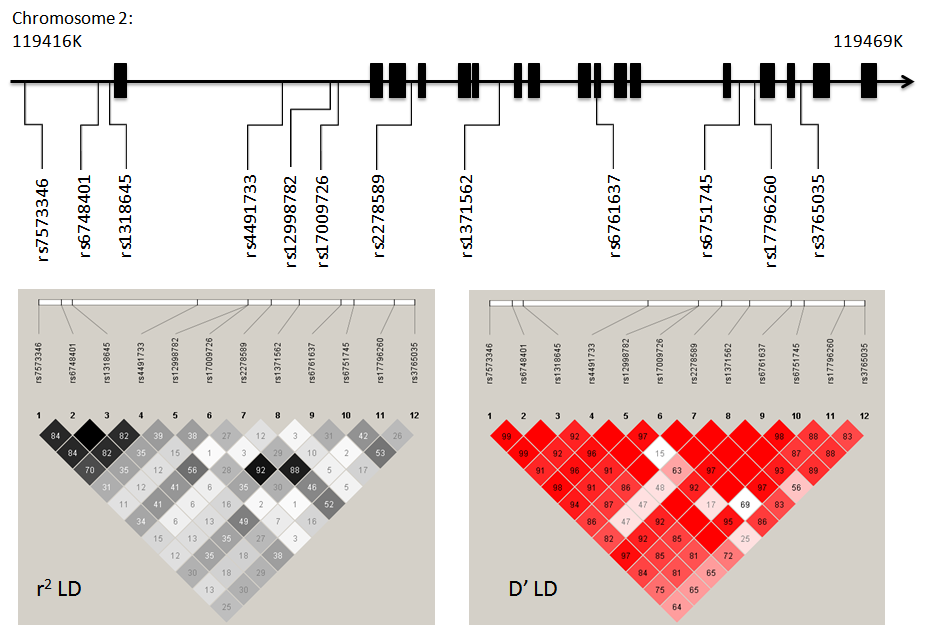


**Figure S2.** **Chromosomal map and linkage disequilibrium of *MARCO* polymorphisms in a Vietnamese cohort**

1. Chromosomal map of polymorphisms in *MARCO*, located on chromosome 2, boxes show exonic regions. (B, C) Linkage disequilibrium values (r^2^ and D') between SNPs were generated by Haploview 4.2 using genotype data from the control population (Vietnamese Kinh). Empty squares indicate complete linkage disequilibrium (r^2^ or D'=1).


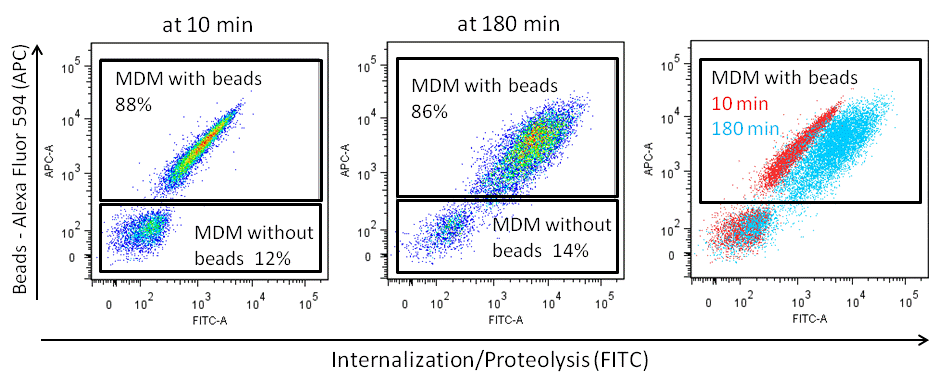


**Figure S3. Assessment of monocyte derived macrophage (MDM) phagocytosis by flow cytometric analysis.** Beads were coated with (1) Alexa Fluor 594 [y axis] and (2) DQ Green BSA substrate (Molecular Probes) [x axis]. Bright green fluorescence is achieved when beads are internalized by MDMs and DQ Green BSA substrate is cleaved by proteolysis to release green fluorescent protein fragments. MDMs were incubated with beads for 10 minutes to allow uptake, then cells were washed three times to remove loosely attached or unbound beads. Green fluorescence was measured at early (10 minutes) and late (180 minutes) time points.
